# Supplementary material for: Time‑resolved multi-omic analysis of paclitaxel exposure in human iPSC‑derived sensory neurons unveils mechanisms of chemotherapy‑induced peripheral neuropathy
Source: Cell Death Dis. 2026 Feb 10;17(1):211. doi: 10.1038/s41419-026-08445-2 (PMC12921266; doi:10.1038/s41419-026-08445-2)
Supplement: Supplementary file 15 — Dataset 1. [file 41419_2026_8445_MOESM15_ESM.zip › CDD_Open_Data/04_RNA_Seq/Timeline_panel_plots_etc_2023-01-23_1609.zip!/Panel_Plots_etc/Evidence_Plots/24h/evidence_plot_standard contrasts_msigdb_reactome_24h_PTX_vs_DMSO_ID4_M27172_Activation_SREBF.pdf]

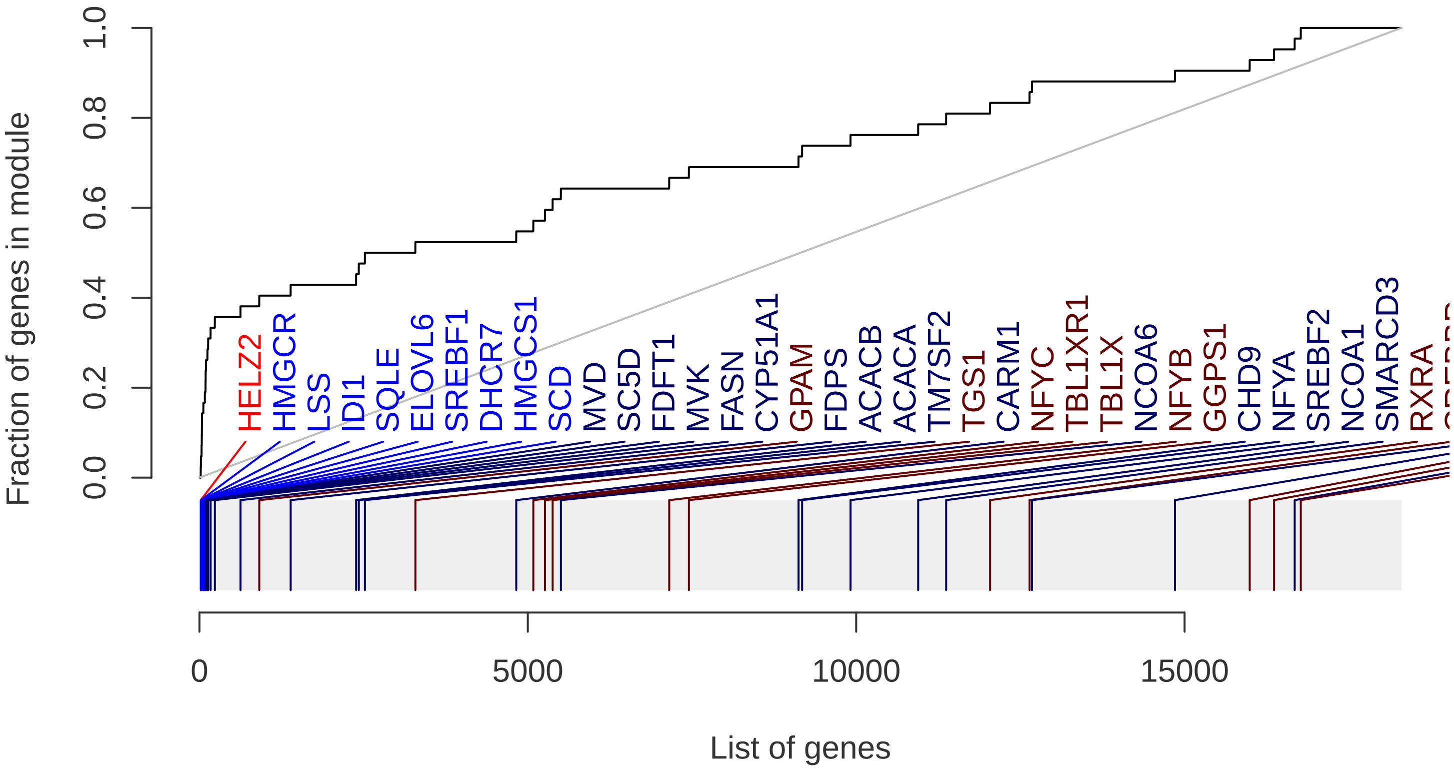
Fraction of genes in module
1.0
0.8
0.6
0.4
0.2
0.0
HELZ2
HMGCR
LSS
IDI1
SQLE
ELOVL6
SREBF1
DHCR7
HMGCS1
SCD
MVD
SC5D
FDFT1
MVK
FASN
CYP51A1
GPAM
FDPS
ACACB
ACACA
TM7SF2
TGS1
CARM1
NFYC
TBL1XR1
TBL1X
NCOA6
NFYB
GGPS1
CHD9
NFYA
SREBF2
NCOA1
SMARCD3
RXRA
0
5000
10000
15000
List of genes
